# Molecular predictors of venous and arterial thrombotic events in patients with myelofibrosis

**DOI:** 10.1007/s00277-025-06361-7

**Published:** 2025-04-27

**Authors:** Olga Morath, Jenny Rinke, Annabell Walter, Carl Crodel, Manja Meggendorfer, Constance Baer, Andreas Hochhaus, Thomas Ernst

**Affiliations:** 1 Klinik für Innere Medizin II, Abteilung Hämatologie und Internistische Onkologie, Universitätsklinikum Jena, Jena, Germany; 2 MLL Münchner Leukämielabor GmbH, München, Germany

**Keywords:** Myelofibrosis, Thrombosis, *DNMT3A*, *JAK2*

## Abstract

While patients with myelofibrosis (MF) face an elevated risk of thrombosis, no validated scoring system currently exists to effectively assess this specific risk. This study aimed to explore distinct molecular risk factors for arterial (ATE) and venous (VTE) thrombosis in a cohort of 141 MF patients. Mutation analysis was performed by next-generation sequencing for a panel of 30 target myeloid genes as previously described: 137 driver and 164 non-driver mutations were detected. *JAK2-V617F* was identified in 77 (55%) patients, *CALR* in 45 (32%) patients, and seven (5%) patients carried an *MPL* variant. Patients #58 and #60 harbored *JAK2-V617F* and *MPL*; and patient #67 was positive for all three driver genes. The *JAK2*-*V617F* variant allele frequency (VAF) was assessed in 66/80 patients, revealing a median of 34.0% (range, 5.0–96.0). *ASXL1* (*n* = 34 patients) were the most common non-driver mutations, followed by *TET2* (*n* = 26), *U2AF1* (*n* = 12), and *DNMT3A* (*n* = 11). During a median follow up of 4.8 years, 24 (17%) patients experienced VTE, 15 (11%) ATE, and two patients experienced both. Among the 24 patients with VTE, 12 (50%) experienced splanchnic vein thrombosis. The *JAK2-V617F* mutation was associated with VTE (OR 2.6, 95% CI 1.01–7.16), while the *DNMT3A* mutation was an independent predictor of ATE (OR 5.40, 95% CI 1.30-22.42). High *JAK2-V617F* VAF (> 50%) was not related with an increased thrombotic risk. Results of this study demonstrate the significance of *DNMT3A* mutations as an independent molecular risk factor for ATE, highlighting the potential to include these somatic non-driver mutations in future thrombosis risk scores.

**Supplementary Information:**

The online version contains supplementary material available at 10.1007/s00277-025-06361-7.

## Introduction

According to the 2022 World Health Organization (WHO) classification for hematopoietic malignancies [1], major categories of *BCR::ABL1*-negative myeloproliferative neoplasms (MPN) include polycythaemia vera (PV), essential thrombocythaemia (ET), and primary myelofibrosis (PMF). Prefibrotic MF (pre-MF) was introduced as a separate entity in the WHO 2016 classification [2]. MF secondary to PV or ET (post-PV MF and post-ET MF) is another disorder that contributes to a large population of patients with MF. MPN are associated with an increased risk of arterial (ATE) and venous (VTE) thromboembolic complications, significantly contributing to the morbidity and mortality [3–5]. Interestingly, the incidence of thrombotic events increases during follow up after the initial diagnosis of MPN, despite the availability of modern cytoreductive therapy [6, 7]. Initially, research on thrombotic risk in patients with MF faced challenges caused by a historical underestimation in comparison to patients with PV and ET, where thrombotic risk scores have been established in patient management guidelines [8, 9]. Although the incidence of thrombotic events in MF, particularly in pre-MF patients may be comparable to those in PV and ET patients [6, 10–14], no score has been developed and validated to assess thrombotic risk in patients with MF.

Furthermore, the role of *JAK2-V617F* mutation presence and variant allele frequency (VAF) in determining thrombotic risk in MF patients remains uncertain. *JAK2-V617F* has been established as a thrombotic risk factor in ET patients for more than a decade [15]. Recent studies have demonstrated a correlation between a high *JAK2-V617F* VAF with an increased thrombotic risk in patients with PV, ET, or in general MPN cohorts [16–22]. However, low *JAK2-V617F* VAF has been predictive of lower survival in PMF [23], implying the presence of a dominant *V617F*-negative clone that provides a more aggressive disease phenotype [24].

Moreover, whether somatic non-driver mutations increase the risk of thrombotic events in MPN, particularly in MF patients, is unknown. Mutations in genes involved in chromosome modification (e.g. *ASXL1* and *EZH2*), epigenetic regulation of DNA methylation (e.g. *DNMT3A* and *TET2*), and RNA splicing (e.g. *U2AF1*, *SF3B1*, *SRSF2*, and *ZRSR2*) are frequently observed in PMF, with some of them being more prevalent than in PV and ET [25–28]. High-risk mutations (i.e. *ASXL1*, *EZH2*, *IDH1/IDH2*, *SRSF2*, and *TP53*) are related to a poorer prognosis and increased rates of leukemic transformation in patients with PMF [29]. Results of recent studies [16, 30], suggest an independent association of the presence of *TET2* or *DNMT3A* mutations with ATE, as well as *ASXL1*, *NRAS*, and *TP53* mutations with VTE in patients in the general MPN cohort. Only a few studies have addressed the association between non-driver mutations and thrombosis in myelofibrosis patients independently of other MPN [31, 32]. Previous findings demonstrated the impact of *TET2* mutations on thrombosis in 228 MF patients in a univariate analysis [32]. Despite these findings, non-driver mutations have not been integrated into widely established thrombotic scoring systems.

Thrombosis assessment tools in patient management guidelines for PV and ET do not distinguish between ATE and VTE thrombosis [8, 9, 33], despite the pathophysiology of ATE and VTE being fundamentally different. A better understanding of risk factors for each thrombosis type is crucial for future risk stratification and preventative recommendations in MPN and, particularly, MF patients. Furthermore, patients with venous thrombosis in unusual sites constitute an additional group with unique molecular characteristics [34]. MPN-associated splanchnic vein thrombosis is frequently a life-threatening complication, with a 5-year mortality estimate of 40% [35].

Therefore, the primary objective of the current study was to identify distinct molecular risk factors for arterial and venous thrombosis in a substantial cohort of MF patients.

## Methods

### Patients

Clinical data of 141 MF patients with biological material available in the biobank and treated between 2005 and 2024 at the Jena University Hospital was analyzed retrospectively.

### Mutation analyses

Mutation analysis was performed for all 141 patients. After erythrocyte lysis, DNA from bone marrow or peripheral blood was isolated using the MagnaPure system (Roche, Mannheim, Germany) or the QIAamp DNA Mini Kit (Qiagen, Hilden, Germany), according to the manufacturers’ recommendations. The hotspot *JAK2-V617F* mutation was analyzed using the amplification-refractory mutation system (*JAK2*-ARMS-PCR, Mastercycler^®^ X50, Eppendorf, Germany) [36].The *JAK2-V617F* VAF was assessed in 66/80 (82.5%) *JAK2*-*V617F* mutated patients by pyrosequencing as described previously [31]. To analyze *CALR* and *MPL* mutations, DNA fragment analysis and Sanger sequencing were performed on the 3500 Genetic Analyzer (Life Technologies, STADT, USA), respectively [37].Next-generation sequencing was performed on the NovaSeq 6000 (Illumina, ILMN, San Diego, CA, USA) after a hybrid capture (IDT Inc. Coralville, IA, USA) or on the NextSeq with the TSCA - TruSeq Custom Amplicon panel (both ILMN). For variant calling either SeqNext 4.3 (JSI Medical Systems, Kippenheim, Germany) or Pisces (version 5.1.3.60, available via ILMN BaseSpace) was used [38].A core set of 30 myeloid leukemia-associated genes was evaluated (Supplement 1).

### Statistics

The primary endpoint was the occurrence of a thrombotic event, acute leukemia transformation or death at any time. VTE include deep vein thrombosis, pulmonary embolism, splanchnic vein thrombosis, and cerebral venous sinus thrombosis. ATE include ischemic stroke or transient ischemic attack, peripheral vascular disease, or acute coronary syndrome. Groups were compared using the Wilcoxon-Mann-Whitney test. Categorical variables were reported as counts and proportions, and compared using chi-square or Fisher’s exact test. Univariable and multivariable logistic regression analyses were used to identify independent predictors of events. Results were presented as odds ratio (OR) with 95% confidence interval (CI). All reported p-values are two-sided, and *p* < 0.05 was considered statistically significant. SPSS version 28 was used for statistical calculations. Patients with unusual site thrombosis were not investigated separately because of the small sample size (*n* = 13).

## Results

In total, 141 patients were investigated: 33 (23%) patients with pre-MF, 77 (55%) with PMF, and 31 (22%) with secondary MF after ET or PV. Pre-MF was only diagnosed in patients after 2016 according to the criteria outlined in the WHO 2016 classification [2]. Follow up period averaged 4.8 years (range, 3.0–7.0 years). At the time of data cutoff, 24 (17%) patients had died. Patients’ characteristics are summarized in Table 1.

**Table 1:** Patients’ characteristics

Characteristics	Cases (%)
Age (years) • median • range	58 22–85
Sex • female • male	60 (42.5%) 81 (57.5%)
MF subgroup • prefibrotic • primary overt fibrotic • post-PV • post-ET	33 (23.4%) 77 (54.6%) 12 (8.5%) 19 (13.5%)
Treatment of MF • watch and wait • ruxolitinib • other JAK inhibitor • hydroxyurea • interferon • clinical trial or other*	20 (14.2%) 77 (54.6%) 8 (5.7%) 15 (10.6%) 9 (6.4%) 12 (8.5%)
Prior history of thrombosis • venous • arterial • both	8 (5.7%) 7 (4.9%) 2 (1.4%)
Antiplatelet therapy at the baseline Anticoagulation at the baseline	55 (39.0%) 24 (17.0%)
Thrombosis as diagnostic event or during follow up • venous • arterial • both	24 (17.0%) 15 (10.6%) 2 (1.4%)
Cardiovascular risk factors • smoking • arterial hypertension • diabetes mellitus • hypercholesterolemia	23 (16.3%) 66 (46.8%) 25 (17.7%) 20 (14.2%)

* patient declined the treatment

### Mutational landscape of MF patients

**Fig. 1:**
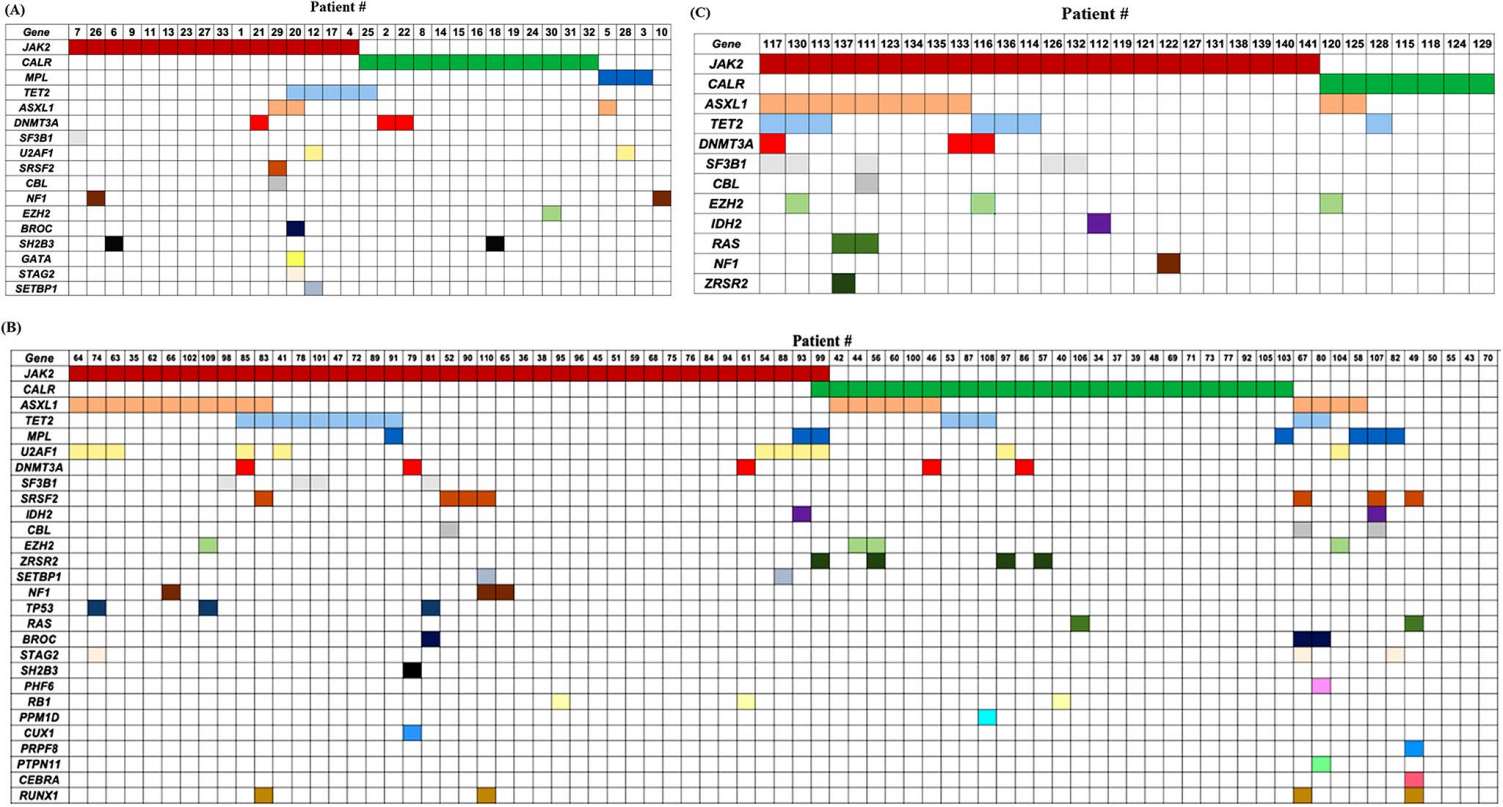
Mutational landscape of pre-MF (**A**), overt MF (**B**) and secondary MF (**C**) patients. In total, 301 individual mutations affecting 30 genes were detected

Mutation analysis was performed for all patients (Fig. 1A-C). In total, 137 driver mutations were observed: *JAK2-V617F* was identified in 77 (55%) patients, *CALR* mutations were detected in 45 (32%) patients, and seven (5%) patients carried an *MPL* variant. Patients #58 and #60 harbored two driver mutations (*JAK2-V617F* and *MPL*); and patient #67 was positive for all three driver genes. Nine (6%) patients were triple-negative. There was no *JAK2* exon 12 mutation. The *JAK2-V617F* VAF was assessed in 66/80 (82.5%) *JAK2*-*V617F* mutated patients, and the median was 34.0% (range, 5.0–96.0). 17/66 (25.8%) patients had a *JAK2*-*V617F* VA*F* > 50%. *JAK2-V617F* VAF did not differ between patients with pre-MF and overt MF. Apart from typical driver mutations, 164 mutations of non-driver genes were found: *ASXL1* (*n* = 34), *TET2* (*n* = 26), *U2AF1* (*n* = 12), *DNMT3A* (*n* = 11), *SF3B1* (*n* = 10), *SRSF2* (*n* = 8), *EZH2* (*n* = 8), *CBL* (*n* = 5), *ZRSR2* (*n* = 5), *NF1* (*n* = 5), *RAS* (*n* = 4), *RUNX1* (*n* = 4), *BROC* (*n* = 4), *SH2B3* (*n* = 4), *SETBP1* (*n* = 3), *TP53* (*n* = 3), *STAG2* (*n* = 3), *PHF6* (*n* = 3), *RB1* (*n* = 3), *IDH2* (*n* = 2), *PPM1D* (*n* = 1), *PTPN1* (*n* = 1), *GATA2* (*n* = 1), *CUX1* (*n* = 1), *PRPF8* (*n* = 1), *CEBPA* (*n* = 1), *KIT* (*n* = 1). Selected mutations comprising the high mutation risk category (any mutations in *ASXL1*,* SRSF2*,* EZH2*,* IDH1*,* IDH2*, and *U2AF1*) [39–43]. were significantly more represented in overt MF in comparison to prefibrotic MF (*p* < 0.001).

### Clinical impact of mutations

Clinical impact of mutations is summarized in Table 2. No association was found between somatic mutations and age (for *ASXL1* mutations *p* = 0.57, *TET2p* = 0.18, *U2AF1p* = 0.19, *DNMT3Ap* = 0.77) or sex (for *ASXL1* mutations *p* = 0.17, *TET2p* = 0.53, *U2AF1p* = 0.11, *DNMT3Ap* = 0.49). Patients with *ASXL1* mutation (*p* = 0.03) and the triple-negative MF (*p* < 0.001) had an increased risk of progressing to secondary acute myeloid leukemia. A history of thrombotic events did not correlate with a poorer prognosis (*p* = 0.39).

**Table 2:** Clinical impact of mutations

Mutations	Clinical impact	*p* value	OR, 95% CI
*JAK2-V617F*	venous thrombosis	0.05	2.6, 1.01–7.16
*DNMT3A*	arterial thrombosis	0.02	5.4, 1.30-22.42
*ASXL1*	progressing to secondary acute leukemia	0.03	2.8, 1.09-6,99

During follow up, 41 (29.1%) patients developed at least one thrombotic event (Table 1). Of the 24 patients who developed VTE at the time of MF diagnosis or during follow up, 12 (50%) had a thrombotic event in the splanchnic area and one patient a cerebral venous sinus thrombosis. There were no significant differences in the rates of VTE between patients with pre-MF and overt MF (18.2% vs. 20.8%, *p* = 0.76) as well as in the risk of ATE (18.2% vs. 7.8%, *p* = 0.11). The multivariable analysis revealed a significant association between *JAK2-V617F* mutation (OR 2.6, 95% CI 1.01–7.16, *p* = 0.05) and an increased risk of developing VTE, but not ATE. The *JAK2-V617F* VAF did not differ between patients with pre-MF and overt MF, and did not affect the risk of VTE (*p* = 0.61).

*DNMT3A* mutation was a significant predictor for ATE (OR 5.4, 95% CI 1.30-22.42, *p* = 0.02) after adjusting for sex, age, and cardiovascular risk factors: smoking, arterial hypertension diabetes mellitus hypercholesterolemia, in the multivariable analysis.

### DNMT3A mutation as an independent molecular risk factor for ATE in MF patients

**Fig. 2:**
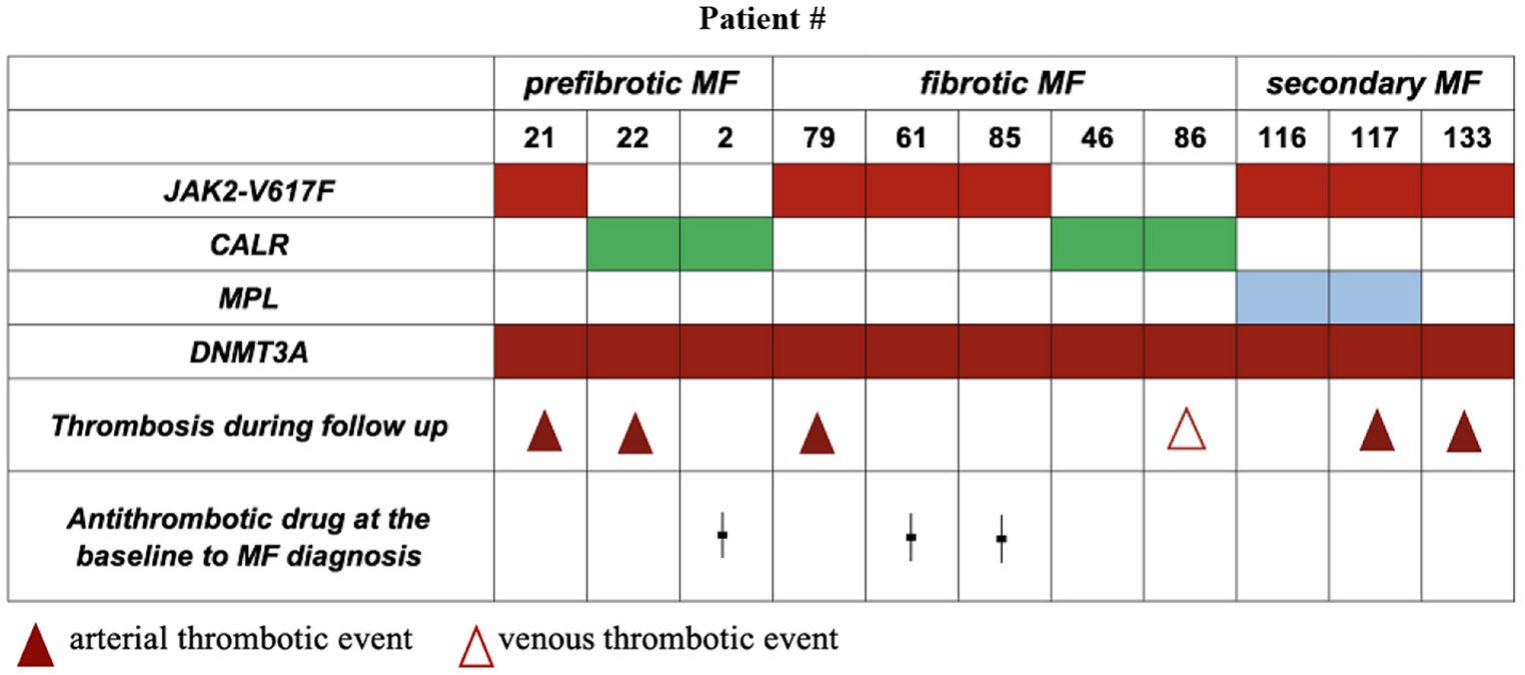
*DNMT3A* mutations and thrombotic events in MF patients

*DNMT3A* mutations were detected in 11 patients: in seven patients as a co-mutation to *JAK2-V617F* (including two patients with *MPL* mutations) and in four patients as a cooperating variant to a *CALR* mutation (Fig. 2). Five patients (#21, #22, #46, #7, and #23) developed an ATE during follow up, and one patient (#53) developed VTE. Three patients (#2, #28, and #52) received at the baseline of MF diagnosis an anticoagulation or antiplatelet drug. The median age of patients with *DNMT3A* mutations was 63.0 years (range, 41.0–85.0). *DNMT3A* VAF analysis was performed in all *DNMT3A* mutated patients (Supplement 2 Table), revealing a median of 41.0% (range, 3.0–44.0).

## Discussion

With this study, the molecular landscape of MF associated with venous and arterial thrombosis was delineated. Compared to previously published data [16, 44–50], a similar incidence of thrombotic events was observed in this cohort. This underscores the importance of comprehensive thrombosis assessment and personalized risk stratification in the management of MF patients.

A total of 301 individual mutations affecting 30 driver and non-driver genes were identified, highlighting the heterogeneous mutational landscape of MF and the dominance of high-risk mutations in patients with overt MF compared to patients with pre-MF, consistent with previous findings [31, 39, 41–43, 51–56]. In the current study, there was no significant difference in the rates of VTE between patients with pre-MF and overt MF (18.2% vs. 20.8%, *p* = 0.76). However, there was a clear trend in the risk of ATE in patients with pre-MF (18.2% vs. 7.8%, *p* = 0.11). Recent studies have similarly revealed an even higher thrombotic incidence in patients with pre-MF [6, 13, 57–59] in comparison with patients with overt MF. The plausible explanation for these findings could be younger age in patients with pre-MF, which might result in less attention paid to cardiovascular risk factors, a later diagnosis of MF, or higher platelet counts and hemoglobin levels [57, 58].

Molecular risk factors for venous and arterial events were examined separately. According to previous investigations [16, 22], the presence of the *JAK2-V617F* mutation was associated with an increased risk of VTE, but had no effect on the risk of ATE. The *JAK2-V617F* mutation encodes an activated tyrosine kinase in the STAT signaling pathway, a regulator of inflammation and myeloproliferation. Experimental studies have demonstrated the interplay between *JAK2* gain-of-function mutations, inflammation, and thrombosis pathogenesis in mice models [60–62] and cell lines [63]. Additionally, hematopoietic *JAK2-V617F* expression may promote venous thrombosis by enhancing the formation of neutrophil extracellular traps within the thrombus [64]. Furthermore, the heightened prevalence of splanchnic venous thrombosis in MPN remains an unresolved question. Previous studies suggested the presence of the *JAK2* mutation in endothelial cells of the splanchnic veins, potentially contributing to the thrombotic activity [65, 66]. According to recent data [48, 50, 67], the current study observed an even higher occurrence of splanchnic vein thrombosis, likely due to the referral of patients with splanchnic vein thrombosis to our specialized center for MPN.

Compared to patients with PV and ET, the *JAK2-V617F* VAF did not correlate with thrombotic risk in the current cohort of MF patients [20, 68]. The most probable reason could be a high incidence of splanchnic vein thrombosis in the current study, in line with MPN patients with atypical thrombosis showing lower *JAK2-V617F* VAF [69–71]. Similar to recent findings [59, 72], we propose that even patients with a low *JAK2-V617F* VAF should be considered at high risk for VTE.

Somatic mutations in *DNMT3A* are commonly identified as clonal hematopoiesis of indeterminate potential (CHIP) in healthy elderly adults without blood cancers [73] and has been associated with cardiovascular events [74–76]. However, the observations that eight out of eleven patients with *DNMT3A* mutations in the current cohort were under the age of 65, and that the *DNMT3A* mutation remained a predictor of ATE, even after adjusting for traditional cardiovascular risk factors and age, show that such epigenetic mutations do not exclusively represent age-related CHIP. This is consistent with a study conducted by Ernst et al. [77] where a surprisingly high frequency of *ASXL1* mutations in young adults with chronic myeloid leukemia was observed, underlining that mutations detected in addition to the known drivers, are not necessarily merely age-related events in MPN.

Only one patient (#23) developed an acute coronary syndrome, whereas the other patients experienced cerebrovascular events. Recent studies have found that the presence of CHIP mutations, specifically *DNMT3A*, is significantly associated with stroke and its severity [78–81]. According to colleagues [82, 83], *DNMT3A* mutations result in an aggressive inflammatory transcriptome, enhance macrophage activity and stimulate proinflammatory T-cell polarization. Further studies in mouse models and cell lines demonstrated oncogenic cooperation between *JAK2-V617F* and *DNMT3A* loss, resulting in activation of inflammatory signaling [84]. Additionally, it could be possible that the order of acquisition of *JAK2-V617F* and *DNMT3A* is crucial in the pathogenesis of thrombosis, similar to a recent study that compared patients with *JAK2* mutation first (“*JAK2*-first patients”) and “*TET2*-first patients” [85]. Based on the results of next-generation sequencing, the order of acquisition can only be inferred indirectly, and further analysis using colony-forming progenitor assays or single cell analyses should be performed in this regard [31]. Further investigation into the pathophysiology of myeloproliferative neoplasms particularly MF, may aid in understanding the molecular mechanisms underlying the cardiovascular complications.

According to published data, the presence of *ASXL1* mutations [42, 86–90] and the absence of any driver mutation [91–93] were linked to an increased risk of progressing to secondary acute myeloid leukemia in the current MF cohort. No correlation was found between a poor prognosis and the occurrence of thrombotic events. It could be explained by an increased rate of sepsis and transformation to secondary myeloid leukemia in the causes of mortality, particularly among younger MF patients [5]. In the current study, the majority of patients (13/24, 54.1%) died from sepsis or refractory secondary leukemia.

Study limitations include the single-center analysis at an academic hospital with challenging clinical cases of myelofibrosis and a high proportion of comorbidities, which may lead to confounding bias. Additionally, only patients with MF and complete mutational analysis were included. Further, patients with splanchnic and non-splanchnic thrombotic events should be analyzed separately to better assess the impact of *JAK2-VAF* and non-driver mutations on thrombotic risk. Though the number of patients with *DNMT3A* mutations was limited, and *JAK2*-*V617F* VAF analysis was not performed in all patients, the study still provides valuable new findings, adding to the expanding knowledge that correlates molecular and clinical data in rare patient populations.

## Conclusion

In conclusion, this study provides novel insights into the heterogeneous molecular landscape underlying thrombotic complications in myelofibrosis. The findings demonstrated the significance of *DNMT3A* mutations as an independent molecular risk factor for arterial thrombosis, highlighting the potential to include this somatic non-driver mutation in innovative thrombosis risk scores. The presence of *JAK2-V617F*, regardless of *JAK2-V617F* VAF, predicts VTE. These findings advance as a signal to future prospective studies on developing new thrombotic assessment scores. It is crucial to validate these observations in a larger patient cohort to confirm their clinical relevance and enhance the robustness of future risk assessment models.

## Electronic supplementary material

Below is the link to the electronic supplementary material.

Supplementary Material 1

## Data Availability

No datasets were generated or analysed during the current study.
