# Supplementary material for: Molecular predictors of venous and arterial thrombotic events in patients with myelofibrosis
Source: Ann Hematol. 2025 Apr 27;104(5):2755–63. doi: 10.1007/s00277-025-06361-7 (PMC12141381; doi:10.1007/s00277-025-06361-7)
Supplement: Supplementary file 1 — Supplementary Material 1 [file 277_2025_6361_MOESM1_ESM.docx]

**Supplement 1** **List** of 30 myeloid genes, included in the next-generation sequencing (NGS) panel.

*JAK2*

*CALR*

*MPL*

*ASXL1*

*BROC*

*CBL*

*CUX1*

*DNMT3A*

*EZH2*

*GATA2*

*GNB1*

*IDH1*

*IDH2*

*KIT*

*KRAS*

*NF1*

*NRAS*

*PHF6*

*PPM1D*

*PTPN11*

*RUNX1*

*SETBP1*

*SF3B1*

*SH2B3*

*SRSF2*

*STAG2*

*TET2*

*TP53*

*U2AF1*

*ZRSR2*

**Supplement 2 Table** Somatic *DNMT3A* mutations in MF patients

|  | **Sex, age** | ***DNMT3A*, variant ensemble** | **VAF, %*** | **Protein** |
| --- | --- | --- | --- | --- |
| #21 | Male, 60 | 23; c.2645G>A | 3 | p.Arg882His |
| #22 | Male, 64 | 18; c.2119G>A | 6 | p.Gly707Ser |
| #2 | Female, 63 | c.1978T>C | 41 | p. Tyr660His |
| #46 | Male, 85 | 12; c.1474+1G>T | 43 | p. splice site mutation |
| #28 | Female, 67 | 9; c.1020T>A | 38 | p.Cys340* |
| #52 | Male, 65 | 23; c.2638del | 3 | p.Met880* |
| #13 | Male, 64 | c. 1667+1G>A | 42 | p. splice site mutation |
| #53 | Female, 49 | 23; c.2645G>A | 21 | p.ARg882His |
| #7 | Female, 41 | c. 682 del | 43 | p.Glu228Lysfs*88 |
| #6 | Female, 44 | c.2644C>T | 44 | p.Arg882Cys |
| #23 | Female, 49 | 19; c.2246G>T | 43 | p.Arg749Leu |

**^*^** *VAF: variant allelic frequency, % of mutated reads compared to the total reads (mutated plus wild type) as determined by next-generation sequencing (NGS). Data was analyzed with SeqNext 4.3 using a 3% sensitivity level (JSI Medical Systems, Kippenheim, Germany)*
